# Video-assisted thoracoscopic surgery using mobile computed tomography: New method for locating of small lung nodules

**DOI:** 10.1186/1749-8090-9-110

**Published:** 2014-06-20

**Authors:** Kazuto Ohtaka, Yasuhiro Takahashi, Kichizo Kaga, Naoto Senmaru, Yoshihisa Kotani, Yoshiro Matsui

**Affiliations:** 1 Department of Thoracic Surgery, Steel Memorial Muroran Hospital, Chiribetsu-cho, 050-0076 Muroran, Hokkaido, Japan; 2 Department of Cardiovascular and Thoracic Surgery, Hokkaido University Graduate School of Medicine, Sapporo, Hokkaido, Japan; 3 Department of Orthopedic Surgery, Steel Memorial Muroran Hospital, Muroran, Hokkaido, Japan

**Keywords:** O-arm, Lung, Video-assisted thoracoscopic surgery, Ground glass nodules

## Abstract

**Background:**

The O-arm is an intraoperative imaging device that can provide computed tomography images. Surgery for small lung tumors was performed based on intraoperative computed tomography images obtained using the O-arm. This study evaluated the usefulness of the O-arm in thoracic surgery.

**Methods:**

From July 2013 to November 2013, 10 patients with small lung nodules or ground glass nodules underwent video-assisted thoracoscopic surgery using the O-arm. A needle was placed on the visceral pleura near the nodules. After the lung was re-expanded, intraoperative computed tomography was performed using the O-arm. Then, the positional relationship between the needle marking and the tumor was recognized based on the intraoperative computed tomography images, and lung resection was performed.

**Results:**

In 9 patients, the tumor could be seen on intraoperative computed tomography images using the O-arm. In 1 patient with a ground glass nodule, the lesion could not be seen, but its location could be inferred by comparison between preoperative and intraoperative computed tomography images. In only 1 patient with a ground glass nodule, a pathological complete resection was not performed. There were no complications related to the use of the O-arm.

**Conclusions:**

The O-arm may be an additional tool to facilitate intraoperative localization and surgical resection of non-palpable lung lesions.

## Background

To perform surgery for small lung tumors that include ground glass nodules (GGNs), preoperative or intraoperative procedures to identify the locations of the tumors are generally needed. The preoperative marking procedures principally consist of two methods: the percutaneous method and the transbronchial method. The percutaneous method involves computed tomography (CT)-guided placement of various marking materials, including hook-wire, vital dye, and radioactive material [1-5]. The transbronchial method involves bronchoscopy-guided placement of various marking materials, including metallic coils and barium [6]. These preoperative marking methods require intraoperative fluoroscopic guidance to localize the marking materials. However, these methods have some disadvantages. The percutaneous method has a limitation with respect to the puncture site, as well as some serious complications (pneumothorax, dissemination, and air embolism) [7]. The transbronchial method sometimes needs advanced bronchoscopy techniques. Additionally, because the lung is collapsed during surgery, intraoperative positional relationships between a marking material and the lesion may differ from the preoperative positional relationships, and it may cause incomplete resection. Other methods to localize the tumors have been reported, such as intraoperative ultrasonography and the intrathoracic stamping method [8-10].

**Figure 1:**
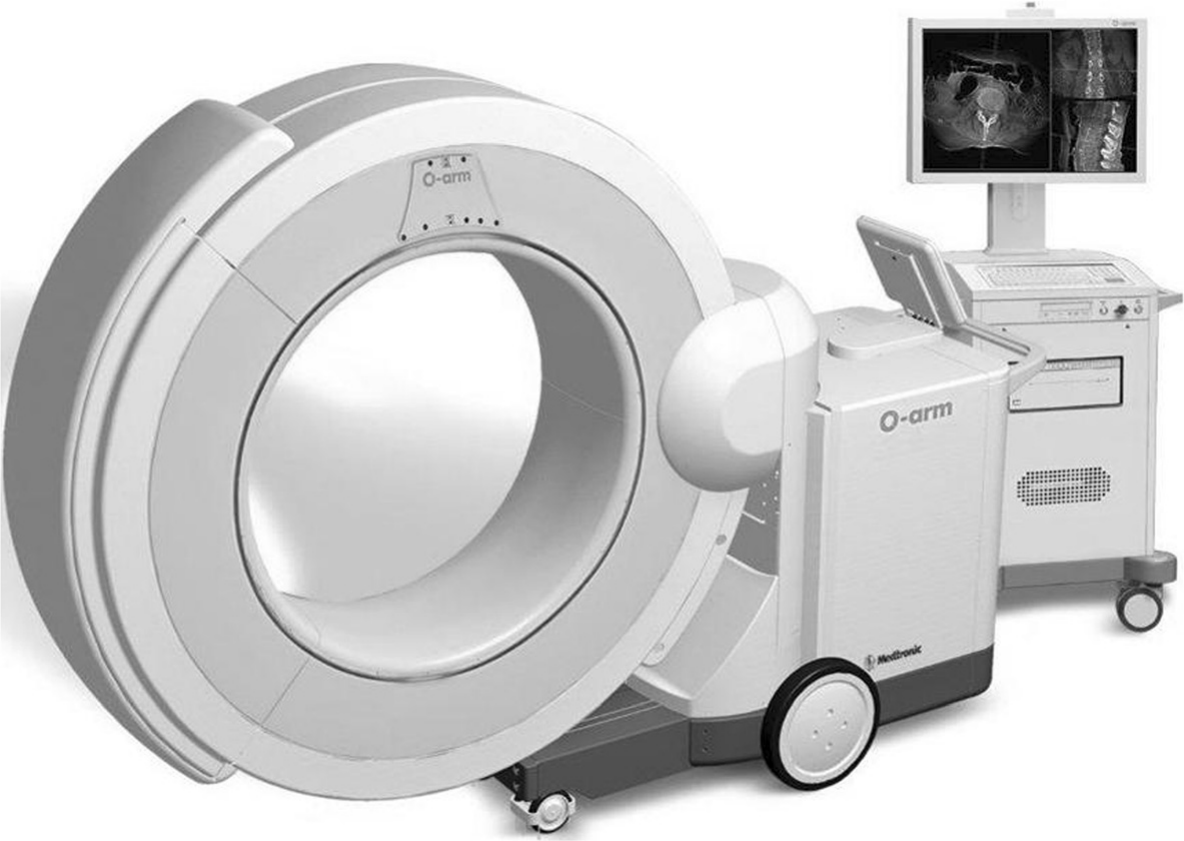
O-arm surgical imaging system.

The O-arm Surgical Imaging System (Medtronic Japan Co., Ltd., Tokyo, Japan) is an intraoperative, full-rotation, multidimensional image system that functions as an intraoperative imaging device with a flat-panel detector that provides two-dimensional (2D) fluoroscopic imaging and three-dimensional (3D) cone-beam CT imaging (Figure 1). This system has been used in spine, orthopedic, and trauma-related surgeries, and has recently also been used in neurosurgery [11-13]. In these surgeries, after the navigation imaging is constructed based on intraoperative CT images using the O-arm, the surgery is performed using these navigation images. Petrov et al. suggested that the O-arm could be used for not only bone tissue but also for soft tissue [14]. Therefore, we hypothesized that the O-arm could be used for small lung tumors.

In this study, surgery for small lung tumors that included GGNs was performed based on intraoperative CT images using the O-arm. This is the first report using the O-arm in thoracic surgery.

## Methods

The Steel Memorial Muroran Hospital Institutional Review Board approved this study. Informed consent was obtained from the patients for the publication of this report and any accompanying images.

### Patients

From July 2013 to November 2013, 23 patients with a small lung tumor underwent video-assisted thoracoscopic surgery (VATS) in Steel Memorial Muroran Hospital. Of these 23 patients, 10 patients with small lung lesions underwent the surgery using the O-arm.

### O-arm

The O-arm Surgical Imaging System was used. This system can provide 2D fluoroscopic imaging and 3D cone-beam CT imaging. Cone-beam CT images are acquired in standard mode (standard definition), where about 400 projection views over 360 degrees are acquired in 13 seconds. The 3D imaging volumes, which have a diameter of 20 cm and a length of 15 cm, are reconstructed with 512 × 512 × 192 voxels (0.830 mm axial, coronal, and sagittal slice thicknesses) in approximately 20 seconds. The dose length projection ranges 140 to 320 mGycm depending on patient size.

### Indication for localization using the O-arm

In our institute, clinical indication for preoperative localization were on the basis of the following preoperative CT findings: (1) maximum diameter of nodule of 10 mm or less, (2) minimum distance between the visceral pleura and superior border of the nodule of 10 mm or more, (3) GGN which had no contact with visceral pleura. So we considered that the O-arm would be eventually used for these lesions. However, because there had been no experience using the O-arm for lung tumors in thoracic surgery, the O-arm was used for small tumors that were predicted to be in easily recognized locations in this study.

### Surgical Technique

Sublober resection was performed by VATS with a 3-cm access thoracotomy incision and 2 ports. When both lobectomy and sublober resection in a different lobe was performed, it was performed by VATS with a 7-cm thoracotomy incision, a 3-cm small thoracotomy incision, and 2 ports.

Under general anesthesia with a double-lumen tube in the lateral position, povidone-iodine was applied to the patient who was wrapped in a sterile drape. The O-arm system was wrapped in a plastic drape and bought into the operation field. The operation table used was a normal type, not a special type.

First, to decrease frequency of the O-arm scan, the needle marking was put on the visceral pleura lying directly on the lesion using the intrathoracic stamping method reported by Kawada et al. [10]. A mark was put on the skin at the shortest distance from the lesion based on preoperative CT images scanned at maximal inspiration. An injection needle was inserted vertically from the skin mark through the chest wall into the pleural cavity. Nylon thread was inserted through an injection needle into the pleural cavity and withdrawn from the pleural cavity through a small thoracotomy. A small gauze ball containing indigo carmine dye (Daiichi Sankyo Co., Ltd., Tokyo, Japan) was tied to the nylon thread and pulled back into the pleural cavity. The gauze ball was tugged towards the internal surface of the thoracic wall, where it was anchored. After the lung was re-expanded, the dye from the gauze ball stamped the visceral pleura. After the lung was then collapsed again, a needle with 4-0 polydiaxonone (PDS) thread was placed near the stamp on the visceral pleura.

Second, the O-arm scan was performed. The O-arm was positioned to the target level of the needle marking based on the 2D fluoroscopic images using the O-arm. After the lung was re-expanded, the O-arm 3D scan was performed at maximal inspiration, and CT images were reconstructed.

Third, the positional relationship between the lesion and the needle marking was determined based on these CT images. If the needle marking was away from the lesion, the needle with 4-0 PDS thread was again placed near the lesion based on the first intraoperative CT images. The O-arm 3D scan was then performed again.

Finally, lung resection was performed based on the intraoperative CT images.

The time for the set-up of the O-arm was about 5 minutes. The time for intraoperative use of the O-arm was about 10 to 15 minutes.

All medical staff in the operation room wore radioprotectors. When the O-arm 3D scan was performed, the anesthesiologist went behind the radioprotective lead screen near the anesthesia equipment, and the radiological technologist and the surgeon went behind the operator’s console, where was expected to have exposure of less than 0.1 mR per spin. Other paramedical staff left the operating room during the O-arm 3D scan and returned immediately after the 3D scan.

## Results

**Figure 2:**
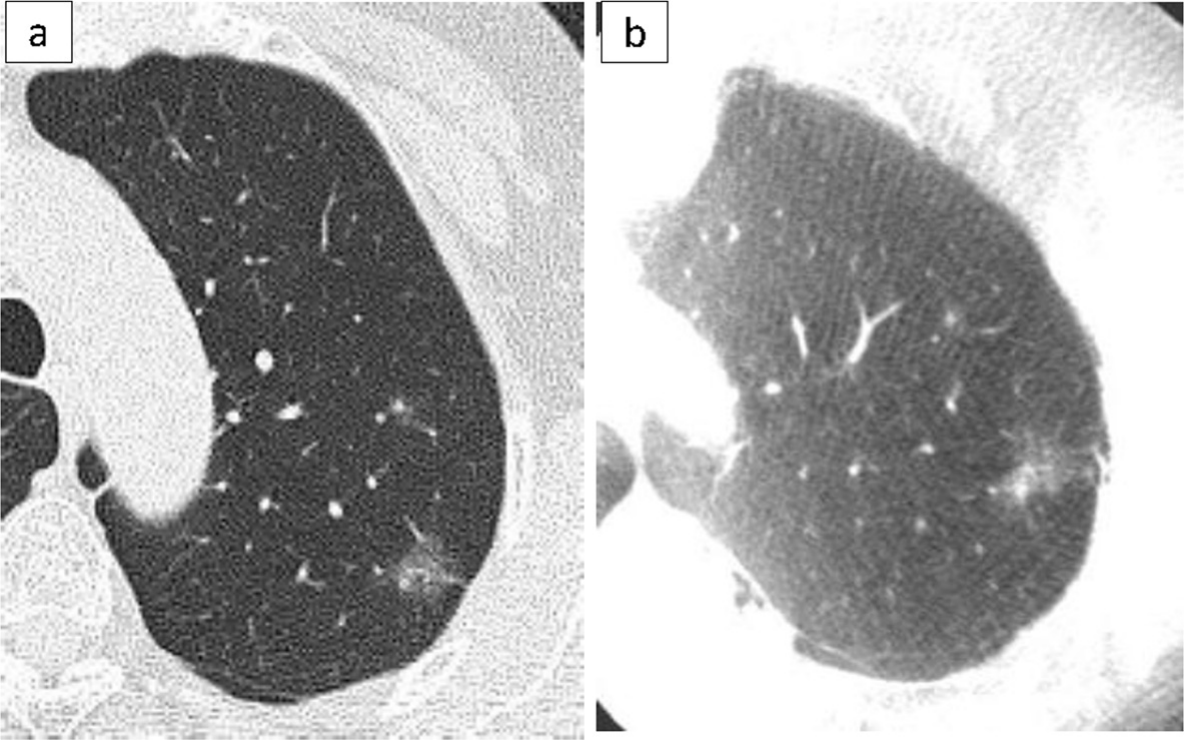
**A GGN with a solid part.** Preoperative CT shows a GGN, 14 mm in diameter, with a small solid part in the left upper lobe **(a)**. The O-arm shows the same finding as the preoperative CT **(b)**.

**Figure 3:**
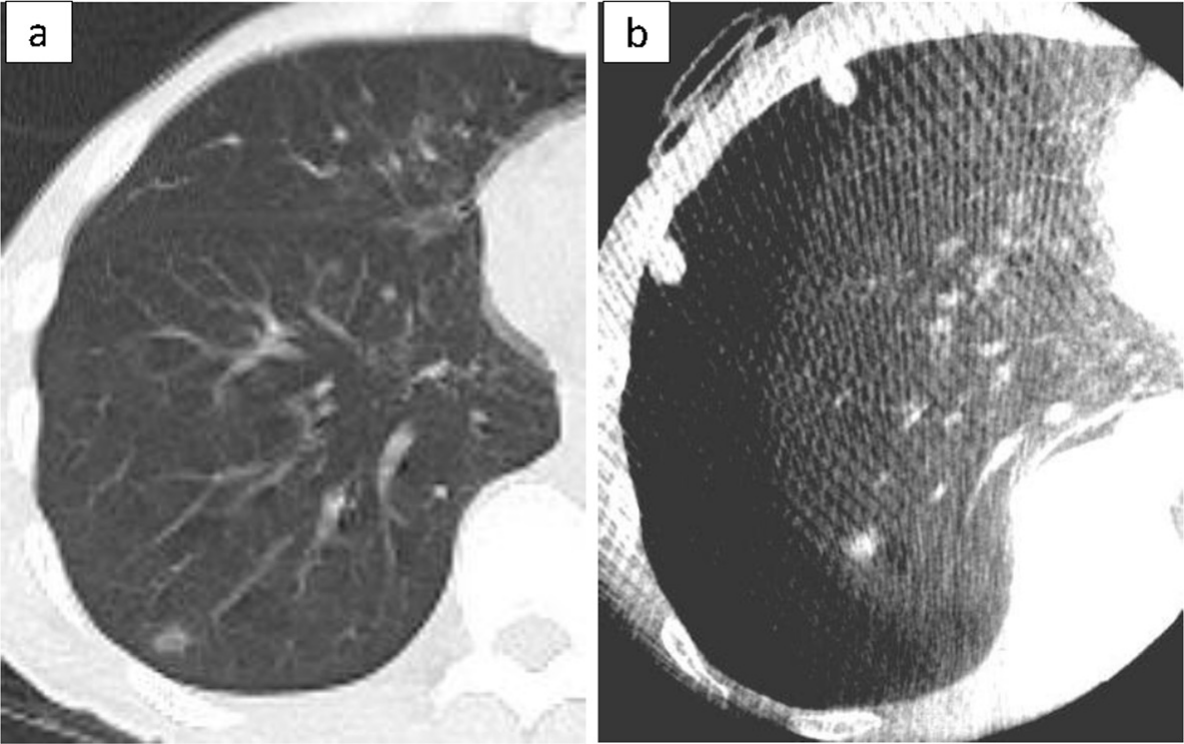
**A GGN without a solid part.** Preoperative CT shows a GGN, 9 mm in diameter, without a solid part **(a)**. When the lung is incompletely re-expanded, the O-arm shows a higher density lesion than the preoperative CT finding, similar to a solid tumor **(b)**.

**Figure 4:**
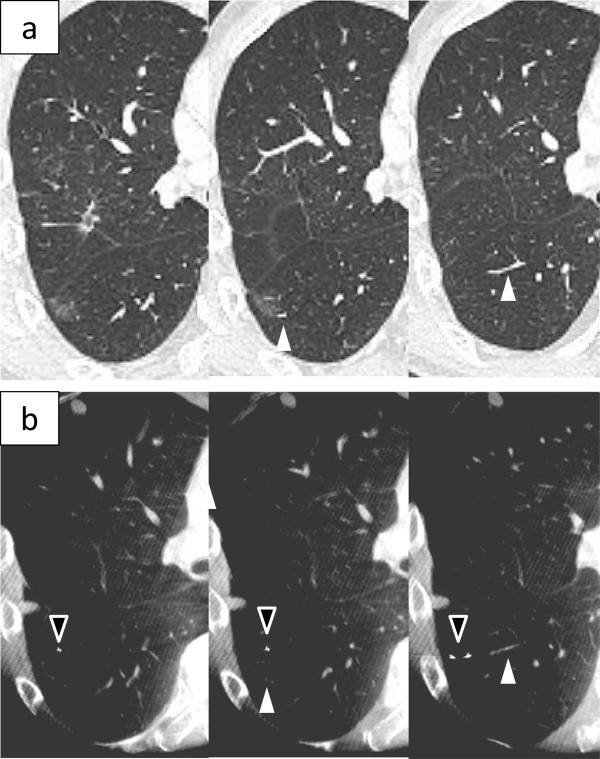
**A GGN without a solid part.** Preoperative CT shows a GGO lesion, 10 mm in diameter, without a solid part **(a)**. The O-arm could not show the lesion. However, the location of the tumor could be inferred by comparing the positions of the peripheral pulmonary vessels (white arrowhead) or the bronchus near the tumor on the preoperative and intraoperative CT images **(b)**. A PDS needle is placed as a marker (black arrowhead).

**Figure 5:**
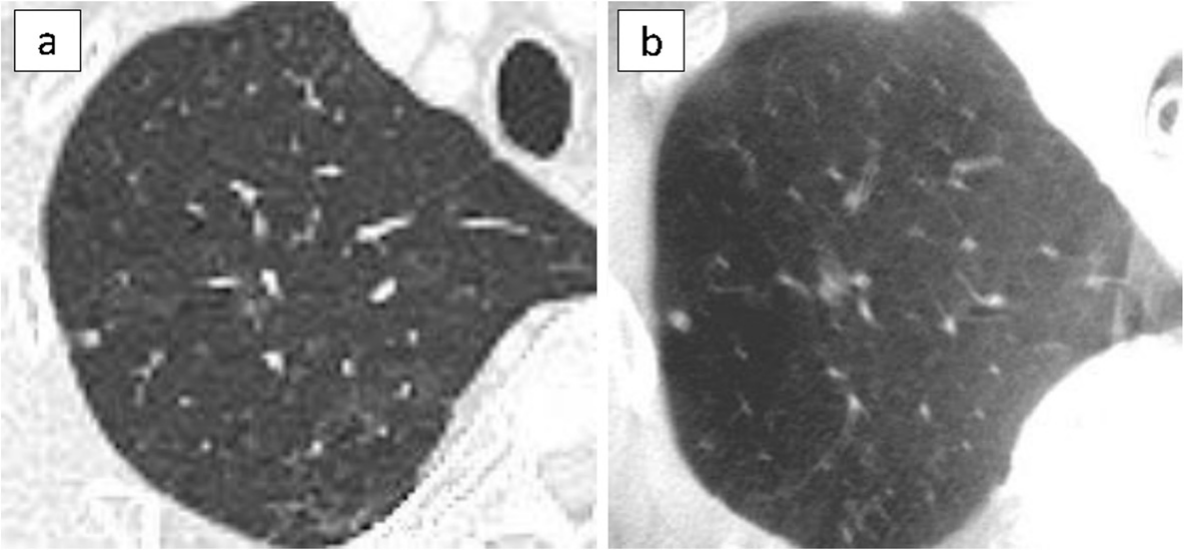
**A small nodule.** Preoperative CT shows a small nodule, 3 mm in diameter **(a)**. The O-arm shows an obvious nodule **(b)**.

The characteristic of the 10 patients are shown in Table 1. The pathological diagnoses were 4 primary cancers, 5 metastatic tumors, and 1 benign tumor. The preoperative CT findings were 5 solid lesions and 5 GGNs. The median size of the resected tumor was 10 mm (range, 6-14 mm). The operative procedures were 8 sublober resections and 2 both lobectomy and sublober resection in a different lobe. Of the 8 patients with sublober resection, 1 patient underwent resection for 6 lesions, 1 patient underwent resection for 2 lesions, and the remaining 6 patients underwent resection for 1 lesion. The median operative time for all 10 patients was 105 minutes (range, 58-243 minutes). The median operative time for the 6 patients with sublober resection of a lesion was 67 minutes (range, 58-110 minutes).In 9 patients, the tumor could be seen on intraoperative CT images using the O-arm (Figure 2). Of them, in 1 patient with a GGN, because the lung was incompletely re-expanded, intraoperative CT using the O-arm showed a higher density lesion than the preoperative CT, similar to a solid tumor (Figure 3).In 1 patient with a GGN, the tumor could not be seen, but its location could be inferred by comparing the positions of the peripheral pulmonary vessels or the bronchus near the tumor on the preoperative and intraoperative CT images (Figure 4). Additionally, a small nodule, even 3 mm in diameter, was accidentally scanned and could be seen obviously by the O-arm (Figure 5).

**Table 1:** Patients’ characteristics

	**N = 10**
Age, median value (range), years	66 (52-80)
Sex, n	
Male/Female	5/5
CT findings, n	
Solid lesions/Ground grass nodules	5/5
Lesion of tumor, n	
Right upper lobe/Right lower lobe	3/3
Left upper lobe/Left lower lobe	3/1
Diagnosis	
Primary cancer/Metastatic tumor/benign tumor	4/5/1
Size of tumor, median value (range), mm	10 (6-14)
Operative procedure, n	
Sublober resection/Lobectomy with sublober resection	8/2
Operative time, median value (range), minutes	106 (58-243)
Blood loss, median value (range), mL	3 (0-100)
Postoperative complication, n	
Pulmonary fistula	3

The O-arm scan was performed 1 time in 4 patients, 2 times in 4 patients, 3 times in the 1 patient in whom the tumor could not be seen on intraoperative CT, and 5 times in the 1 patient who underwent resections for 6 lesions.

In 9 patients, pathological complete resection was achieved; in only 1 patient with a GGN, a pathological complete resection was not achieved. This might have been caused by a technical error during resection. This patient underwent additional resection later.

There were no complications related to the use of the O-arm.

## Discussion

Surgery for small lung tumors that included GGNs was performed based on intraoperative CT images using the O-arm. In our experience, a small nodule, 3 mm in diameter, could be easily seen on intraoperative CT images using the O-arm. Thus, the O-arm may be able to show even very small solid tumors. A GGN could be obviously seen on intraoperative CT images using the O-arm. A GGN without a solid part could not be seen in 1 patient. However, the location of the tumor could be inferred by comparing the positions of the peripheral pulmonary vessels or the bronchus near the tumor on the preoperative and intraoperative CT images. Because the O-arm can provide much the same CT images as the preoperative CT images, the location of the tumor can be inferred by comparison between preoperative and intraoperative CT images if the tumor is not shown on intraoperative CT images using the O-arm. Additionally, using the O-arm when the lung was incompletely re-expanded, a GGN could be shown as a higher density lesion than on the preoperative CT images. The O-arm may make it possible to perform surgery for small, non-palpable lung lesions.

In our experiences, only 1 patient with a GGN could not be seen the tumor on the intraoperaive CT images. This might have been due to technical difficulties with scan conditions. Based on our experiences using the O-arm, if the O-arm was positioned under the condition that the tumor was located in the center of the O shape arm, the tumor could be obviously seen on the intraoperative CT images. However, if the O-arm was positioned under the condition that the tumor was not located in the center of the O shape arm, the tumor might not be seen. After we experienced the patient who the tumor could not be seen on the intraoperative CT images, we adjusted the O-arm position under the condition that the tumor was located in the center of the O shape arm and did not experience that case.

There are several benefits in surgery using the O-arm. First, it is not necessary to perform any type of preoperative marking procedure. Preoperative marking procedures, such as the percutaneous method and the transbronchial method, can be difficult for patients and can cause serious complications as pneumothorax, dissemination, and air embolism that may result in the delay or cancellation of surgery [7]. Second, there is a limit to the tumor locations that can be marked with preoperative marking procedures, but there is no such limit with the O-arm.

The disadvantage in surgery using the O-arm is the exposure to radiation. Medtronic Inc. has reported that the effective whole body dose using the O-arm standard 3D protocols for the chest was lower than for 8, 16, and 64-slice CT [15]. In a report dealing with orthopedic surgery, the mean radiation dose of the O-arm 3D scan was comparable to that of half the dose of a 64 multislice CT scan [16]. We estimated that the O-arm resulted in lower radiation exposure than conventional CT in thoracic surgery. However, when it is compared to preoperative marking method, it remains unclear which has higher total radiation exposure for patients.

In our experience, a needle marking was put on the visceral pleura around the tumor based on preoperative skin marking, which was placed based on preoperative CT images. If the preoperative skin marking was not near the tumor, the first needle marking on the visceral pleura was also not near the tumor, and intraoperative CT scanning using the O-arm was needed more than once. To decrease the frequency of intraoperative CT scans and radiation exposure, it is necessary to place the skin marking with more precision.

To reduce radiation exposure for medical staff, when the O-arm scan was performed, medical staffs wearing a radioprotector went behind the radioprotective lead screen or got away from the O-arm. In a report involving orthopedic surgery, the O-arm resulted in less radiation exposure to the surgeon than the C-arm [17]. We considered that there was no major problem with radiation exposure for medical staff when appropriate measures were used with the O-arm.

## Conclusions

This is the first report of the use of the O-arm—which can be used to perform intraoperative CT and has been used in orthopedic surgery—in thoracic surgery. The O-arm may be an additional tool to facilitate intraoperative localization and surgical resection of non-palpable lung lesions.

## Abbreviations

GGN: Ground glass nodule; VATS: Video-assisted thoracoscopic surgery.

## Competing interests

The authors declare that they have no competing interests.

## Authors’ contributions

KO performed the study design, the acquisition of data, and the analysis, and drafted the manuscript. YT, KK, NS,YK and YM helped to draft the manuscript. All authors read and approved the final manuscript.
